# Echocardiographic reference values in elderly with a focus on octogenarians and older

**DOI:** 10.1016/j.ijcha.2026.101872

**Published:** 2026-01-17

**Authors:** Miguel Quintana, Mira Carling, Anders Olofsson, Dag Isaksson, Jenny Fahlen, Elin Kärrman, Fredrik Pihl, Roland Forsberg, Björn Persson, Alen Lovric, Thomas Gustafsson, Karin Bouma

**Affiliations:** a Karolinska Institutet, Institution of Laboratory Medicine, Stockholm, Sweden; b Department of Clinical Physiology, Karolinska University Hospital, Sweden; c Department of Clinical Physiology, Östersunds Hospital, Östersund, Sweden

**Keywords:** 2D echocardiography, Reference values, Elderly individuals, Healthy

## Abstract

**Aims:**

Most published material on echocardiographic reference values includes individuals up to the age of 60–70, but reference values for older individuals remain scarce. Accurate interpretation of transthoracic echocardiographic reference values in octogenarians and older individuals requires updated values that reflect healthy ageing. This study aims to compare transthoracic echocardiographic reference values of healthy octogenarians and older individuals with those of younger age groups.

**Methods and results:**

A total of 248 individuals were studied. The group was divided into three age categories: sexagenarians, septuagenarians, and octogenarians or older. The participants underwent a standard transthoracic echocardiographic examination according to current guidelines. The main differences between age groups were observed in values related to left ventricular diastolic function. There was a significant difference in some values related to systolic function, such as a significant decrease in mitral annular plane systolic excursion in octogenarians compared to sexagenarians and septuagenarians.

**Conclusion:**

The present study showed statistically significant differences in some echocardiographic parameters primarily reflecting the left ventricular diastolic function. In addition, a significant difference in some values related to systolic function were also found. These findings emphasize the need for age-adapted reference values to improve diagnostic accuracy in elderly.

## 1 Introduction

The average age of today’s population is steadily increasing, as is the incidence and prevalence of heart disease. Currently, heart disease occurs in 50% of people over 60, and 85% of people over 80 [1]. Most publications on echocardiographic reference values include individuals up to the age of 60–70 years, but reference values for older individuals are still rare [2], [3], [4], [5].

Standard two-dimensional (2D) and motion mode (M-Mode) echocardiography are the most widely used imaging techniques for assessing structure and function of the heart. More advanced assessment of left ventricular (LV) function has become possible by pulsed wave (PW) and tissue Doppler imaging (TDI) techniques. This is especially useful when assessing diastolic function, as it is more complex than assessing systolic function and as it may be impaired before the onset of global or regional LV systolic dysfunction [6].

The ageing heart undergoes anatomical and physiological changes to maintain adequate contractile LV function, collectively referred to as cardiac remodeling. There are two types of cardiac remodeling: physiological remodeling, as seen in pregnancy or endurance training, where contractile function is normal or increased; and pathological remodeling, which occurs in conditions such as hypertension, myocardial infarction (MI), and advancing age. The initial changes are protective and preservative but eventually lead to myocardial stiffening and fibrosis, resulting in impaired diastolic and systolic function [7]. Differentiating physiological from pathological remodeling is especially challenging in elderly individuals, as age-associated changes are difficult to separate from pathological changes due to cardiovascular disease. Without reliable reference values for this group, it’s even more challenging. Cardiac remodeling can be difficult to detect, but various echocardiographic modalities can reveal changes in morphology and function across different age groups.

### 1.1 Earlier similar studies

Several studies on echocardiographic reference values have been published [2], [3], [4], [5]. However, these studies include few octogenarians or older individuals. For example, the NORRE study included participants aged 25–75 years (2), the WASE study included those aged 18–65+ years (3), and the EchoNoRMAL study included individuals up to 80 years (4). The latter two studies included octogenarians, but the categorization of age groups was unclear. In the EchoNoRMAL study, octogenarians were categorized as “70”, and in the WASE study as “65+” [3], [4]. Finally, the HUNT study included an “80+” category, but with only 24 individuals, resulting in low statistical power and uncertain interpretation [5]. Therefore, a knowledge gap exists when reference values are applied in a clinical setting.

From 2016 to 2020, 17% of echocardiographic examinations in the Department of Clinical Physiology, Östersund Hospital, and 12% in the Department of Clinical Physiology, Karolinska University Hospital, Stockholm, were performed on people aged 80 and over. Current reference values may not reflect the actual cardiac health of this age group.

## 2 Hypothesis

Echocardiographic reference values in octogenarians and older will differ from those in sexagenarians and septuagenarians.

## 3 Aim

The aim of this study was to compare transthoracic echocardiographic reference values of standard M-mode, 2D, PW Doppler and TDI of healthy elderly individuals in different age-groups.

## 4 Methods

### 4.1 Study population

The echocardiographic data was collected between 2018 and 2022. The study population was recruited either by advertisement or from patients referred for echocardiographic examination at Östersund Hospital, Department of Clinical Physiology. Octogenarians and older participants were mainly recruited through advertisements, due to difficulties in finding healthy patients over 80 years referred to echocardiography. The study included 248 individuals without diagnosed heart disease. These were further divided into three age groups: Group A (60–69 years) n = 91, Group B (70–79 years) n = 86, and Group C (80 + years) n = 71.

### 4.2 Inclusion/exclusion criteria

Inclusion criteria were absence of heart diseases or other diseases that might affect the function or structure of the heart, see Table 1.

**Table 1:** Exclusion criteria.

Exclusion criteria	
1. Heart diseases	2. Vascular diseases
**1.1 Known coronary artery disease**	**2.1** Known peripheral vascular diseases requiring surgical or percutaneous vascular intervention
**1.2 Cardiomyopathies**	
Hypertrophic	
Dilated	***3. Other medical conditions***
Arrhythmogenic	**3.1** Renal failure on dialysis, pre- dialysis stages or after kidney transplantation
Non-Compaction	
Tako-Tsubo	
**1.3 Pericardial diseases**	**3.2** Severe liver failure with signs of portal hypertension or investigation for liver transplantation
**1.4 ECG abnormalities**	
Atrio-ventricular block (degree II or more)	
Left bundle branch block	**3.3** Acute pulmonary embolism during the last 3 months
Pathological Q-waves	
Signs of left ventricular hypertrophy confirmed by echocardiography	**3.4** On/after treatment with chemotherapeutic agents or radiotherapy of the left side of the thorax
Permanent atrial fibrillation	
Paroxysmal atrial fibrillation	
**1.5 Valvular heart disease of more than** **Mild degree**	**3.5** Type 2 Diabetes Mellitus
	**3.6** Arterial hypertension
**1.6 Cardiac implantable devices**	
Permanent atrial and/or ventricular pacing	
Implantable cardiac defibrillator (ICD)	

### 4.3 Methods

The comprehensive transthoracic echocardiographic examinations were performed by an experienced biomedical scientist and post-acquisition analysis were performed by a senior physician, specialist in echocardiography (blinded to the participants age) during the years 2018–2022 at Östersund Hospital. The final analysis was done by the senior physician. The ultrasound machines used were Siemens Acuson SC2000, probes 4V1C and 4Z1C (Siemens, California, USA). All patients were examined with M-mode, 2D echocardiography, PW Doppler, and TDI according to current guidelines [8]. All examinations were performed with participants in the left lateral decubitus position. Video clips containing three consecutive heart cycles were acquired during end expiration.

### 4.4 Population demographics and biological data

The demographic and blood test data collected for each participant are shown in Table 2.

**Table 2:** Population demographics and blood test data.

Demographic and blood test data	
Demographics	Blood tests
Age (years)	Haematocrit (%)
Gender	Haemoglobin (g/L)
Height (cm)	Creatinine (mmol/L)
Weight (kg)	Glomerular Filtration (mL/min/1.7)
Body surface area (BSA)	Na (mmol/L)
Body mass index (BMI)	K+ (mmol/L)
Heart rate (bpm)	Glucose (mmol/L)
Systolic blood pressure (mmHg)	Total Cholesterol (mmol/L)
Diastolic blood pressure (mmHg)	HDL-Cholesterol (mmol/L)
	LDL-Cholesterol (mmol/L)
	Triglyceride (mmol/L)

### 4.5 Echocardiographic variables

M-Mode echocardiography was used to measure mitral annular plane systolic excursion (MAPSE) at the LV inferior, anterior, lateral and septal wall. It was also used to measure tricuspid annular plane systolic excursion (TAPSE).

2D echocardiography was applied for measurements of the aortic dimensions, LV outflow tract (LVOT), septal wall thickness (SWT), posterior wall thickness (PWT), LV end-systolic diameter (LVESD), LV end-diastolic diameter (LVEDD) and right ventricular mid diameter (RVm). Left atrial area (LAarea) and volume (LAV), right atrial area (RAarea) and volume (RAV) were obtained. LV end-systolic volume (LVESV) and LV end-diastolic volume (LVEDV) were measured using the Simpson Biplane method, with LVEF calculated accordingly.

PW Doppler was recorded at the LVOT and right ventricular outflow tract during end-expiration. Mitral inflow velocities (E and A waves) were obtained with the sample volume positioned at the tips of the mitral valve leaflets. TDI was measured at the lateral (lat) and septal (sep) annulus of the mitral valve to obtain s′, e′, and a′ velocities, and at the tricuspid annulus for the same parameters.

### 4.6 Statistics

The mean and standard deviation (SD) of continuous variables were calculated for all groups for each of the measured variable. GraphPad Prism version 10.1.2 was used for statistical analyses. Comparisons between the three age-groups were done with a single-factor analysis of variance (ANOVA) test, and T-tests (two-sample assuming equal variances) to determine statistical differences between the age groups. Statistical significance was set to p-value < 0.05.

### 4.7 Consent

This study is part of the “Octogenarian Population Ultrasound Screened” (OctoPUS) study and approved by the local ethical committee (Dnr 2020–00363). All participants have given written consent to participate in the study.

## 5 Results

Demographic properties are presented in Table 3. Group A had significantly higher weight and height than Group B (p = 0.02 and p = 0.03) and Group C (p < 0.001 and p < 0.001). Consequently, Group A had a higher body surface area (BSA) than Groups B and C. There was no significant difference in blood pressure or heart rate between the groups.

**Table 3:** Demographic properties.

Group properties	A (n = 91)	B (n = 86)	C (n = 71)	A vs B	A vs C	B vs C
	Mean ± SD			p-values		
Percentage of females, %	52	59	60			
Weight, kg	80 ± 16.7	72 ± 15.8	68 ± 11.0	0.02	< 0.001	NS
Height, cm	171 ± 13.9	168 ± 14.5	166 ± 9.7	0.03	< 0.001	NS
BSA, m^2^	1.9 ± 0.2	1.8 ± 0.2	1.8 ± 0.2	< 0.001	< 0.001	NS
BMI	27 ± 4.0	25 ± 4.0	25 ± 3.0	NS	< 0.001	NS
SBP, mmHg	132 ± 17.2	134 ± 15.9	137 ± 17.3	NS	NS	NS
DBP, mmHg	78 ± 9.4	79 ± 7.8	80 ± 8.0	NS	NS	NS
HR, bpm	70 ± 11.7	72 ± 10.5	70 ± 8.9	NS	NS	NS

Data presented as Mean ± SD. NS = non-significant.

Variables obtained from 2D, M-mode, PW Doppler, TDI, and semi-automatic LVEF are presented in Table 4, Table 5, Table 6. LVEDD was larger in group A than in group B (p = 0.05) and group C (p = 0.003). RVm and SWT were larger in group C than in group A (p < 0.001 and p = 0.005, respectively) and group B (p < 0.001 and p = 0.004, respectively). RAarea was smaller in group C than in group A (p = 0.004) and group B (p = 0.03). MAPSE displacement at the septum was lower in group C than in group B (p = 0.01) and group A (p < 0.001). Similar findings were observed in the inferior wall, where MAPSE was lower in group C than in group A (p < 0.001) and group B (p = 0.01). TAPSE was higher in group A than in group B (p = 0.04) and group C (p = 0.02).

**Table 4:** 2D and M-Mode variables.

	A (n = 91)	B (n = 86)	C (n = 71)	A vs B	A vs C	B vs C
Variable	Mean ± SD			p-values		
Aortic Annulus, mm	26 ± 3.6	26 ± 3.2	26 ± 4.4	NS	NS	NS
Aortic Root, mm	32 ± 4.3	32 ± 3.3	33 ± 4.1	NS	NS	NS
Aortic STJ, mm	29 ± 3.9	28 ± 3.2	29 ± 3.9	NS	NS	NS
Aorta Ascendens, mm	33 ± 3.9	33 ± 3.6	33 ± 4.3	NS	NS	NS
LVOT, mm	21 ± 2.2	20 ± 2.1	21 ± 2.2	NS	NS	NS
Aortic Arch, mm	28 ± 3.2	28 ± 2.9	28 ± 3.3	NS	0.01	NS
LVEDD, mm	44 ± 5.7	42 ± 5.2	42 ± 4.2	0.05	0.003	NS
LVESD, mm	29 ± 5.3	28 ± 4.5	28 ± 4.8	NS	0.03	NS
RVm, mm	37 ± 4.6	37 ± 3.9	40 ± 3.8	NS	< 0.001	< 0.001
SWT, mm	10 ± 1.4	10 ± 1.2	11 ± 1.4	NS	0.005	0.004
PWT, mm	9 ± 1.3	9 ± 1.3	10 ± 1.4	NS	NS	0.02
LA area, cm²	21 ± 3.5	20 ± 4.5	19 ± 4.4	NS	NS	NS
LA area, cm²/m^2^	11 ± 1.6	11 ± 2.0	11 ± 2.2	NS	NS	NS
LAV, mL	58 ± 18.4	57 ± 19.8	56 ± 18.8	NS	NS	NS
LAV, mL/m^2^	30 ± 9.0	31 ± 9.7	32 ± 9.7	NS	NS	NS
RAarea, cm²	18 ± 3.6	18 ± 3.5	17 ± 3.4	NS	0.004	0.03
RAarea, cm²/m^2^	9 ± 1.7	10 ± 1.7	9 ± 1.8	NS	NS	NS
RAV, mL	52 ± 17.2	49 ± 16.7	44 ± 14.8	NS	0.004	NS
RAV, mL/m^2^	26 ± 8.7	27 ± 8.2	25 ± 8.4	NS	NS	NS
TAPSE, mm	26 ± 4.1	24 ± 3.6	24 ± 4.0	0.04	0.02	NS
MAPSE-s, mm	13 ± 2.1	12 ± 2.5	11 ± 2.6	NS	< 0.001	0.01
MAPSE-l, mm	15 ± 2.5	14 ± 2.5	14 ± 3.0	NS	0.02	NS
MAPSE-i, mm	14 ± 2.1	14 ± 2.3	13 ± 2.8	0.02	< 0.001	0.01
MAPSE-a, mm	14 ± 2.7	13 ± 3.0	13 ± 3.5	0.008	NS	NS
MAPSE, mm	14 ± 1.8	13 ± 2.0	13 ± 2.5	NS	NS	NS

Data presented as Mean ± SD. NS = non-significant.

***Aortic STJ***: Aortic Root Diameter at Sinus Tubularis Junction; ***LA area***: Left atrial area; ***LAV***: Left atrial volume; ***LVEDD***: Left ventricular end diastolic diameter; ***LVESD***: Left ventricular end systolic diameter; ***LVOT***: Left Ventricular Outflow Tract diameter; ***MAPSE***: Mitral Annulus Plane Systolic Excursion (average of all walls); ***MAPSE-a***: Mitral Annulus Plane Systolic Excursion at Anterior Wall; ***MAPSE-i***: Mitral Annulus Plane Systolic Excursion at Inferior Wall; ***MAPSE-l***: Mitral Annulus Plane Systolic Excursion at Lateral Wall; ***MAPSE-s***: Mitral Annulus Plane Systolic Excursion at Septum; ***PWT***: End-diastolic posterior wall thickness; ***RAarea***: Right atrial area; ***RAV***: Right atrial volume; ***RVm***: Right ventricular mid-diameter; ***SWT***: End-diastolic septal wall thickness; ***TAPSE***: Tricuspid Annulus Plane Systolic Excursion.

**Table 5:** PW Doppler and TDI variables.

	A (n = 91)	B (n = 86)	C (n = 71)	A vs B	A vs C	B vs C
Variable	Mean ± SD			p-values		
RVS Pressure, mmHg	22 ± 4.8	23 ± 5.7	23 ± 5.0	NS	NS	NS
E-Velocity, m/s	0.69 ± 0.1	0.66 ± 0.1	0.62 ± 0.2	NS	NS	NS
A-Velocity, m/s	0.73 ± 0.2	0.76 ± 0.2	0.81 ± 0.2	NS	NS	NS
E dec. time, ms	215 ± 39.1	230 ± 47.3	246 ± 66.9	0.02	< 0.001	0.05
E slope	3.32 ± 1.0	3.14 ± 1.7	2.68 ± 1.1	NS	< 0.001	0.05
Mitral PHT, ms	61.4 ± 14.2	66.8 ± 13.8	69.8 ± 22.4	0.01	0.004	NS
E/A	0.96 ± 0.2	0.89 ± 0.2	0.80 ± 0.3	0.03	< 0.001	0.02
PV Systolic Velocity, m/s	0.67 ± 0.1	0.68 ± 0.1	0.65 ± 0.1	NS	NS	NS
PV Diastolic Velocity, m/s	0.48 ± 0.1	0.46 ± 0.1	0.46 ± 0.1	NS	NS	NS
PV A Velocity, m/s	0.83 ± 4.4	0.41 ± 0.2	0.4 ± 0.1	NS	NS	NS
PV A Duration, ms	125 ± 17.3	128 ± 18.7	126 ± 19.6	NS	NS	NS
e' sep, cm/s	8.49 ± 1.8	8.23 ± 5.9	6.32 ± 1.4	0.02	< 0.001	0.006
e' lat, cm/s	10.61 ± 2.2	9.10 ± 2.4	7.56 ± 2.1	< 0.001	< 0.001	< 0.001
a’sep, cm/s	12.80 ± 9.1	10.77 ± 2.5	10.65 ± 2.1	NS	NS	NS
a' lat, cm/s	12.04 ± 9.4	11.41 ± 3.3	10.74 ± 2.5	NS	NS	NS
s' sep, cm/s	9.48 ± 1.6	9.03 ± 6.5	10.97 ± 1.1	NS	NS	NS
s' lat, cm/s	10.07 ± 2.4	9.89 ± 2.6	8.78 ± 2.1	NS	< 0.001	0.001
E/é-s	7.84 ± 2.8	8.71 ± 2.6	10.13 ± 2.8	0.04	< 0.001	0.001
E/é-l	6.72 ± 1.9	7.56 ± 2.6	8.8 ± 3.2	0.02	< 0.001	0.009
E/é	7.28 ± 2.1	8.14 ± 2.4	9.46 ± 2.7	0.01	< 0.001	0.001
RVe’, cm/s	0.14 ± 0.02	0.13 ± 0.03	0.12 ± 0.03	NS	NS	NS
RVa’, cm/s	0.18 ± 0.04	0.16 ± 0.04	016 ± 0.03	NS	0.02	NS
RVs’, cm/s	0.15 ± 0.03	0.15 ± 0.03	0.14 ± 0.03	NS	NS	NS

Data presented as Mean ± SD. NS = non-significant.

***A-Velocity***.: Mitral inflow A-wave velocity;***a’lat****:* a’ wave velocity, lateral wall*;****a’se*p**: a’ wave velocity septal wall; ***E wave dec. time***: E-wave deceleration time; ***E/A***: Ratio between early (E) and late (A) mitral inflow velocities; ***E/é***: The average of the ratio E/é at lateral and septal walls; ***E/é-l***: The ratio of E/é at lateral wall; ***E/é-s***: The ratio of E/é at septal wall; ***e’lat***: e’wave velocity, lateral wall; ***e’sep***: e’wave velocity, septal wall; ***E-Velocity:*** Mitral inflow E-wave velocity; ***Mitral PHT***: Mitral pressure half-time; ***PV A duration***: Pulmonary vein atrial flow duration; ***PV A velocity***: Pulmonary vein atrial flow velocity; ***PV Diastolic velocity***: Pulmonary vein diastolic velocity; ***PV Systolic velocity***: Pulmonary vein systolic velocity; ***RVS Pressure***: Right ventricular systolic pressure; ***RVa’***: Right ventricular a’wave velocity; ***RVe’***: Right ventricular e’wave velocity; ***RVs’***: Right ventricular s’wave velocity; ***s’lat***: Systolic wave velocity, lateral wall; ***s’sep***: Systolic wave velocity, septal wall.

**Table 6:** Semi-Automatic LVEF echocardiographic variables.

	A (n = 91)	B (n = 86)	C (n = 71)	A vs B	A vs C	B vs C
Variable	Mean ± SD			p-values		
LVEDV-A4Ch, ml	88 ± 21.3	79 ± 19.9	77 ± 22.4	0.005	0.001	NS
LVEDV-A4Ch, ml/m^2^	46 ± 8.9	43 ± 8.5	43 ± 10.6	NS	NS	NS
LVESV-A4Ch, ml	38 ± 10.9	34 ± 10.8	33 ± 10.5	0.03	0.009	NS
LVESVix-A4Ch, ml/m^2^	19 ± 5.0	19 ± 4.9	18 ± 5.1	NS	NS	NS
LVSV-A4Ch, ml	51 ± 13.4	45 ± 12.5	44 ± 13.5	NS	NS	NS
LVEF-A4Ch, %	58 ± 6.6	57 ± 6.3	57 ± 6.2	NS	NS	NS
LVEDV-A2Ch, ml	86 ± 21.0	74 ± 22.1	74 ± 22.0	< 0.001	< 0.001	NS
LVEDV-A2Ch, ml/m^2^	44 ± 8.6	41 ± 9.7	41 ± 10.7	0.009	NS	NS
LVESV-A2Ch, ml	36 ± 11.8	32 ± 11.6	32 ± 11.6	0.04	0.03	NS
LVESV-A2Ch, ml/m^2^	19 ± 5.3	18 ± 5.2	18 ± 5.7	NS	NS	NS
LVSV-A2Ch, ml	50 ± 13.0	42 ± 13.1	42 ± 12.9	< 0.001	< 0.001	NS
LVEF-A2Ch, %	59 ± 7.6	57 ± 7.8	58 ± 7.0	NS	NS	NS
LVEDV, ml	87 ± 19.6	76 ± 19.7	76 ± 21.5	< 0.001	< 0.001	NS
LVEDV, ml/m^2^	45 ± 7.7	42 ± 8.3	42 ± 10.1	0.008	NS	NS
LVESV, ml	37 ± 10.3	33 ± 10.7	33 ± 10.4	0.02	0.01	NS
LVESV, ml/m^2^	19 ± 4.8	18 ± 4.8	18 ± 5.0	NS	NS	NS
LVSV, ml	50 ± 11.9	43 ± 11.3	43 ± 12.3	< 0.001	< 0.001	NS
LVEF, %	58 ± 5.8	57 ± 6.5	57 ± 5.4	NS	NS	NS

Data presented as Mean ± SD. NS = non-significant. All variables were calculated according to the Simpson Biplane Method.

***A2C:*** Apical two-chamber view; ***A4C:*** Apical four-chamber view; ***LVEDV:*** Left ventricular end diastolic volume; ***LVEF:*** Left ventricular ejection fraction; ***LVESV:*** Left ventricular systolic function; ***LVSV:*** Left ventricular stroke volume.

Regarding PW Doppler variables: The E-wave deceleration time progressively increased in all groups, with significant differences between groups A and B (p = 0.02), A and C (p < 0.001), and B and C (p = 0.05). The E-wave slope progressively decreased in groups B and C compared to group A (A vs C, p < 0.001; B vs C, p = 0.05). The E/A ratio significantly decreased between groups A, B, and C (A vs B, p = 0.03; A vs C, p < 0.001; B vs C, p = 0.02). Mitral pressure half-time was shorter in group A compared to B and C, with significant differences between groups A and B (p = 0.01) and A and C (p = 0.004).

TDI variables showed that é sep and é lat progressively decreased across the three groups. É sep was higher in Group A than in Group B (p = 0.02) and Group C (p < 0.001). A significant difference was also found between Group B and Group C (p = 0.006). É lat showed significant differences between Group A and Group B, Group A and Group C, and Group B and Group C, with p < 0.001 for all three comparisons. Significant differences were observed in ś lat between Group A and Group C (p < 0.001) and between Group B and Group C (p = 0.001). E/é-Sep, E/é-Lat, and their average (E/é) progressively increased from Group A to Group C (A vs B, p = 0.01; A vs C, p < 0.001; B vs C, p = 0.001) (Table 5).

Table 6 summarizes all semi-automatic LVEF measurements. LV volumes decreased with higher age, although after BSA normalization the significant differences disappeared.

## 6 Discussion

The aim of the study was to compare transthoracic echocardiographic reference values of standard M-Mode, 2D, PW Doppler and TDI of healthy elderly individuals with a focus on octogenarians and older. Results from the present study partly confirm the hypothesis, with significant differences in some variables between groups A, B, and C. The major difference is observed in variables obtained by PW Doppler and TDI, where several variables indicate a progressive decrease in LV diastolic function in older individuals.

Detection of LV diastolic dysfunction is crucial, as it is typically affected before the onset of LV systolic dysfunction. Considering the E/A ratio, the NORRE study showed that in the over 60 group, the total mean E/A ratio was 0.98 ± 0.3. In the present study the 60–69 age group had similar results, while the E/A ratio for octogenarians and older was 0.80 ± 0.3 [9]. Regarding TDI variables, in the NORRE study, e’sep in the over 60 group was 7.6 ± 2.3. In our study, e’sep was similar in groups A and B, but lower in group C (6.32 ± 1.4). For e’lat, NORRE reported a value of 9.6 ± 2.3 in the over 60 group, with the present study showing similar values in sexagenarians, but 7.6 ± 2.1 in octogenarians and older.

Another clinically relevant variable is E/é. The NORRE study reported a value of 8.3 ± 2.2 in the > 60 group, compared to the present study, where sexagenarians had a value of 7.3 ± 2.1 and octogenarians and older had 9.5 ± 2.7. This corresponds well with the current ASE/EACVI guidelines on diastolic function for groups A and B. However, these guidelines do not include octogenarians and older. For instance, the reference ranges for E/A are > 0.8 to < 2.0, >7 cm/s for e’sep, and > 10 cm/s for e’lat [10]. This becomes problematic in a clinical setting, as people above 80 doesn’t have clear cut-off values for evaluation of diastolic function, when their ratio is only comparable to reference values of younger age groups. This emphasises the need for reference values for octogenarians and older in the guidelines.

There were no significant differences in most variables reflecting LV systolic function when comparing septuagenarians to octogenarians, which supports the preservation of LV systolic function with increasing age [11]. In the present study, MAPSE at the septal and inferior walls was lower in octogenarians and older individuals. Our results confirm the findings of a study by Hu et al. [12], which discusses how MAPSE reflects impaired longitudinal displacement and can complement LVEF, as an indicator of early systolic dysfunction. Another study by Emilsson et al. [13] shows that MAPSE decreases with advancing age while EF remains unchanged, which also supports the findings of the present study. This suggests that early systolic dysfunction and stiffening of the heart can be observed in octogenarians and older individuals.

Weight and BSA were higher in the 60–69-year group than in the 70–79 and 80 + groups. Although obesity was not excluded and can affect echocardiographic parameters through its effects on cardiac structure (such as LV hypertrophy, LA enlargement, and increased RV mass) [14], [15] only 22 of 248 participants (<10%) had a BMI > 30 kg/m^2^, making a meaningful impact on study outcomes unlikely.

Chronic obstructive pulmonary disease (COPD) was not excluded. COPD is associated with higher MI risk and with structural RV changes such as reduced TAPSE [16], [17]. In our study, no participant had abnormal TAPSE, suggesting a low likelihood of COPD influence, though it remains a possible confounder.

### 6.1 Strengths and limitations

The main strength of the study is the large number of octogenarians and older participants included. None of the previously mentioned studies [2], [3], [4], [5] included as many individuals in this age group. As the population of octogenarians and the elderly is increasing and is at higher risk of developing cardiac disease, it is a crucial group to study. Another strength is that all echocardiographic examinations were performed at the same hospital by the same biomedical scientists and reviewed by the same physician. This increases reliability, as all examinations were conducted according to the same protocol. In contrast, many similar studies are multi-center studies conducted in different countries with various vendors and technicians, making it substantially more difficult to ensure low variability between technicians and equipment.

One limitation of the study is the definition of healthy individuals among octogenarians and older participants. Simply because the study participants do not have a diagnosis of a cardiac condition or conditions affecting the heart (according to the inclusion and exclusion criteria), we cannot assume that this population is completely healthy. For example, the presence of paroxysmal and asymptomatic atrial fibrillation could not be ruled out for obvious reasons; therefore, the absence of atrial cardiomyopathy and, consequently, heart failure with preserved ejection fraction cannot be guaranteed. Another issue is the lower weight and body surface area in individuals over 70 years compared to younger individuals, which could be associated with sarcopenia and, in turn, with the presence of heart failure with preserved ejection fraction [18], [19].

One limitation of echocardiography is its dependence on the user’s experience, leading to variability in measurements between technicians. For example, the present study found no significant difference in aortic measurements between age groups, whereas similar studies have shown that aortic diameter increases with age [8]. This discrepancy may be due to variation between the technicians performing the echocardiography.

Echocardiographic reference values have been shown to differ between males and females, primarily in structural parameters (2D and M-mode), with women tending to have smaller aortic diameters, LV dimensions, and volumes [2]. As the present study did not stratify values by sex – since the aim was to investigate age-related differences – there may have been underestimation of structural measurements. However, most differences observed in the present study were in Doppler parameters, which have been reported to be less influenced by sex [9], [20].

Since the study was conducted at a single center with predominantly Caucasian individuals, its generalizability is limited, as reference values have been shown to vary between different ethnicities, as demonstrated in the WASE study [3].

### 6.2 Clinical/practical applications

Our study indicates that an update of current guidelines regarding diastolic function is needed, as otherwise this could lead to overdiagnosis of healthy elderly individuals with LV diastolic dysfunction. It is unclear whether the reference values for octogenarians in the cited studies [2], [3], [4], [5] reflect the reality of octogenarians and older, given that these studies primarily focused on individuals younger than 80 years. Therefore, new studies that include the appropriate population are necessary. It is also important to acknowledge the present absence of guideline-based diastolic function classification in the age-group 80 years and older, something that should be established when interpreting these new reference values.

It is well known that octogenarians are highly represented in various cardiac interventions. For example, 14% of percutaneous coronary interventions at Karolinska University Hospital and 20% at Östersunds Hospital during 2016–2020 were performed in individuals over 80 years old. Cardiac surgeries have declined since the introduction of transcatheter aortic valve implantations, in which individuals above 80 are highly represented, accounting for 59% of procedures nationwide in Sweden in 2024 [21]. Taking this into consideration, new echocardiographic reference values will help optimize pre- and post-assessment of these patients, as well as diagnose and optimize treatment for elderly patients with heart failure.

### 6.3 Future studies

As the present study forms part of the larger OctoPUS study, which includes additional data from 3D echocardiography and strain, several studies are planned to analyze myocardial deformation across different age groups and sexes. These studies will provide further information about LV systolic function. Although our study has recruited the largest number of individuals aged 80 and over, we must acknowledge that the total number of participants is still not sufficient to ensure the generalizability of our results. Future larger studies are needed, to compare reference values between males and females, and to include several ethnic groups.

## 7 Conclusions

This study presents echocardiographic reference values for healthy individuals, with a focus on octogenarians and older. The main finding is a significant difference in certain left ventricular diastolic parameters between age groups, indicating a progressive decrease in left ventricular diastolic function in older age groups. In addition, a significant difference between age groups in parameters reflecting LV systolic longitudinal displacement was also observed. This finding may complement LVEF, as an indicator of early systolic dysfunction. Based on these findings, we strongly recommend updating current guidelines to improve diagnostic accuracy for octogenarians.

## Authors contributions

***Concept and design*:** Miguel Quintana, Anders Olofsson, Dag Isaksson, Mira Carling, Thomas Gustafsson, Karin Bouma.

***Acquisition, analysis, or interpretation of data*:** Miguel Quintana, Anders Olofsson, Dag Isaksson, Jenny Fahlen, Elin Kärrman, Fredrik Pihl, Roland Forsberg, Björn Persson, Alen Lovric, Mira Carling, Karin Bouma.

***Drafting of the manuscript*:** Miguel Quintana, Anders Olofsson, Dag Isaksson, Mira Carling, Thomas Gustafsson, Karin Bouma.

***Critical revision of the manuscript for important intellectual content*:** Miguel Quintana, Mira Carling, Thomas Gustafsson, Karin Bouma.

***Statistical analysis*:** Alen Lovric, Miguel Quintana, Mira Carling, Karin Bouma, Thomas Gustafsson.

***Supervision*:** Miguel Quintana, Thomas Gustafsson, Karin Bouma.

## Credit authorship contribution statement

**Miguel Quintana:** Writing – review & editing, Writing – original draft, Supervision, Project administration, Methodology, Investigation, Formal analysis, Conceptualization. **Mira Carling:** Writing – original draft, Supervision, Methodology, Formal analysis, Conceptualization. **Anders Olofsson:** Methodology, Investigation, Formal analysis, Conceptualization. **Dag Isaksson:** Supervision, Methodology, Investigation, Formal analysis. **Jenny Fahlen:** Visualization, Software, Methodology, Investigation, Formal analysis. **Elin Kärrman:** Visualization, Resources, Methodology, Investigation, Formal analysis. **Fredrik Pihl:** Resources, Project administration, Methodology, Investigation, Formal analysis. **Roland Forsberg:** Resources, Project administration, Methodology, Investigation, Formal analysis. **Björn Persson:** Software, Project administration, Methodology, Investigation, Formal analysis. **Alen Lovric:** . **Thomas Gustafsson:** Writing – review & editing, Supervision, Software, Resources, Project administration, Methodology, Investigation, Formal analysis. **Karin Bouma:** Writing – review & editing, Supervision, Methodology, Investigation, Formal analysis, Conceptualization.

## Declaration of competing interest

The authors declare that they have no known competing financial interests or personal relationships that could have appeared to influence the work reported in this paper.
